# Rapid development and mass production of SARS-CoV-2 neutralizing chicken egg yolk antibodies with protective efficacy in hamsters

**DOI:** 10.1186/s40659-024-00508-y

**Published:** 2024-05-06

**Authors:** Binan Zhao, Haoran Peng, Yanjing Zhang, Jie Zhang, Desheng Kong, Sai Cao, Yan Li, Dan Yang, Chuanwen Sun, Xinyi Pu, Ping Zhao, Yan Xu, Kai Zhao, Liangzhi Xie

**Affiliations:** 1 Sinocelltech Ltd, No.31 Kechuang 7th Street, BDA, Beijing, 100176 China; 2 Shanghai Bio-full Biotech Co.,Ltd, Shanghai, 201106 China; 3 Department of Microbiology, Second Military Medical University, Shanghai, 200433 China; 4 Beijing Antibody Research Key Laboratory, Sino Biological Inc, Building 9, Jing DongBei Technology Park, No.18 Ke Chuang 10th St, BDA, Beijing, 100176 China; 5 Beijing Engineering Research Center of Protein and Antibody, Sinocelltech Ltd, No.31 Kechuang 7th Street, BDA, Beijing, 100176 China; 6 College of Life Sciences, Shanghai Normal University, Shanghai, 200234 China; 7 Cell Culture Engineering Center, Chinese Academy of Medical Sciences & Peking Union Medical College, Beijing, 100005 China

**Keywords:** SARS-CoV-2, IgY, Neutralizing assay, Animal experiment, Spray, Passive immunity

## Abstract

Despite the record speed of developing vaccines and therapeutics against the SARS-CoV-2 virus, it is not a given that such success can be secured in future pandemics. In addition, COVID-19 vaccination and application of therapeutics remain low in developing countries. Rapid and low cost mass production of antiviral IgY antibodies could be an attractive alternative or complementary option for vaccine and therapeutic development. In this article, we rapidly produced SARS-CoV-2 antigens, immunized hens and purified IgY antibodies in 2 months after the SARS-CoV-2 gene sequence became public. We further demonstrated that the IgY antibodies competitively block RBD binding to ACE2, neutralize authentic SARS-CoV-2 virus and effectively protect hamsters from SARS-CoV-2 challenge by preventing weight loss and lung pathology, representing the first comprehensive study with IgY antibodies. The process of mass production can be easily implemented in most developing countries and hence could become a new vital option in our toolbox for combating viral pandemics. This study could stimulate further studies, optimization and potential applications of IgY antibodies as therapeutics and prophylactics for human and animals.

**Supplementary Information:**

The online version contains supplementary material available at 10.1186/s40659-024-00508-y.

## Introduction

A novel coronavirus related to severe acute respiratory syndrome (SARS) [1], namely SARS coronavirus 2 (SARS-CoV-2), causes the highly contagious respiratory coronavirus disease 19 (COVID-19).Unfortunately, if infected patients cannot mount an efficacious adaptive immune response or lack the necessary medical treatment for viral clearance to prevent adverse symptoms, they may deteriorate into severe acute respiratory distress syndrome with hypoxemic respiratory failure, resulting in severe pneumonia and multi-organ failure, and even death, within days [2–4].

Vaccination is the most effective measure to control pandemic viral transmission. Fortunately, with the global efforts, multiple COVID-19 vaccines have been developed in record speed and over 5 billion people have been vaccinated with at least 1 dose of vaccine globally [5]. It only took just a couple of months for the mRNA based vaccines approval for clinical trial, but we should not take it for granted that the success achieved with the COVID-19 vaccines can be easily replicated for future emerging pandemic viruses. In fact, vaccine development typically takes 8–10 years or more, and to date, no vaccines have been approved for marketing for HIV, Zika, etc. Although mRNA vaccines have been shown to be highly efficacious and generally safe in clinical trials and mass vaccination, stringent requirements for storage and shipment under sub-zero degree conditions, costs of supply and administration, needle phobia and some rare side effects have led to vaccine hesitancy and lower vaccination in many countries. Alternative prophylactics without injection site or systemic side effects could potentially help to persuade unvaccinated population to receive vaccination.

Chemical antiviral drugs and neutralizing antibodies have shown protective efficacy in treating mild to moderate COVID-19 patients [6–8]. Oral chemical drugs can be taken at home in comparison to the hospital administration of i.v. infused antibody drugs, but chemical antiviral drugs in general could take longer time to develop than monoclonal antibodies. Despite the success achieved with COVID-19 therapeutics at unprecedented speed, there is no certainty that such success can be replicated for future pandemics. In addition, both small molecule drugs and monoclonal antibodies are subject to resistant mutations. Hence, rapid development of more therapeutic options is important to encounter viral mutations.

Egg yolk IgY antibodies can be rapidly produced at relatively low cost. IgY antibodies do not interact with human Fc receptors and do not induce antibody-dependent enhancement or activate human immune rejection [9], and have been used against bacterial and viral infections in humans and animals [10]. Apparently, egg yolk antibody could be an attractive choice for treating infectious diseases such as COVDI-19.

In this article, a proof-of-concept study is carried out to demonstrate feasibility of rapid development of egg yolk antibodies as a COVID-19 prophylactic and therapeutic option.

## Methods

### Cell and viruses

RBD-mFc protein (Cat. 40,592-V05H) were purchased from Sino Biological, Inc. (Beijing, China). The HEK293 cell line, pseudovirus PSV-Luc-Spike (M), and the ACE2-overexpressing 293FT (293FT-ACE2) cell line were provided by Sinocelltech Ltd (Beijing, China). The SARS-CoV-2 isolate Wuhan-Hu-1(GenBank accession no. 622,319), Omicron BA.2.76(EPI_ISL_12810061) and Vero E6 cells were provided by the biosafety level 3 laboratory of the Second Military Medical University (Shanghai, China).

### Immunogen RBD-mFc protein expression, purification, and quality control

The target genes encoding the RBD protein of the SARS-CoV-2 spike protein (Arg319-Phe541; GenBank accession no. YP_009724390.1) were amplified by PCR, subcloned into the vector, and the plasmid DNA was extracted and sequenced to confirm the correct insertion sequence. The protein was expressed in the HEK293E cells through transfection with transfection reagent TF1 (Sino Biological Inc.), and purified protein was processed from the medium supernatant after 7 days. The obtained protein samples were monitored with SDS-PAGE.

### ELISA determination of purified RBD-mFc protein binding activity with ACE2

ELISA was performed to detect the binding of RBD-mFc protein to ACE2 receptor, as previously described [11]. The recombinant RBD-mFc protein was diluted to 2 µg/mL in phosphate-buffered saline (PBS, pH 7.0) and 100 µL was added into each well in 96-well plates. The plate was covered and incubated at 4℃ overnight, then aspirated and washed once with phosphate-buffered saline with 0.05% Tween 20 (PBST). Next, 300 µL blocking solution (2% bovine serum albumin [BSA] in PBST, pH 7.4) was added to each well, incubated at room temperature for 60 min, then aspirated and washed twice. Human recombinant ACE2 protein (His tag) (Cat. 10,108-H08H, Sino Biological) was diluted to 20 µg/mL in dilution buffer (0.1% BSA in PBST, pH 7.4). A 7-point curve was prepared using 5-fold serial dilutions stating from 20 µg/mL. The dilution buffer was used as the zero standard (0 µg/mL). The sample (100 µL) was transferred to each well and the plate was covered and incubated at room temperature for 60 min before it was aspirated and washed three times. Anti-His tag antibody (HRP) (Cat. 105,327-MM02T-H, Sino Biological, Inc.) was diluted to 0.12 µg/mL in dilution buffer (0.5% BSA in PBST, pH 7.4) and 100 µL was transferred to each well. The plate was covered and incubated at room temperature for 60 min, then 200 µL tetra-methyl benzidine (TMB) was transferred to each well. The plate was covered again and incubated at room temperature for 20 min. H_2_SO_4_ (50 µL, 2 M) was added to each well to halt the TMB reaction. The optical density at 450 nm (OD 450 nm) was detected. Data analysis was carried out with GraphPad Prism 8.0.1. The value of EC_50_ was calculated from the best-fit curves for experiment using Prism EC_50_ curve-fitting algorithm.

### RBD-mFc protein immunization of hens

As the immunogen, pure RBD-mFc protein was combined and emulsified in equal proportions with Freund’s immunological adjuvant. The first immunization used complete Freund’s immune adjuvant while the remaining immunizations used incomplete Freund’s immune adjuvant. Each hen (Ten 5-month-old Kangle chickens, weighing about 1600 g; Eggs laid before immunization were collected as a control.) was injected at multiple sites (i.m.) with 300 µg recombinant spike protein and immunized at 10-day intervals for the first five immunizations and then at 1-month intervals afterwards. We collected the eggs, and the egg supernatant titer was tested after a week off per vaccination. The egg yolks were separated, diluted with ultrapure water at 1:7 volume, 1 M HCl solution was added to adjust the pH to 5.0, and then incubated overnight at 4℃ or stored at -20 °C until used. After 30-min centrifugation at 4120 ×*g*, the egg supernatant titer was tested by ELISA.

### IgY production and purification

We used an improved extraction, namely, lipoprotein was first separated at -20℃, then the antibodies were purified using 2-step ammonium sulfate precipitation. The details are as follows: the yolk was carefully removed from the egg white and rolled on a paper towel to remove any egg white before being diluted eight folds with double-distilled water (ddH_2_O) (pH 5.0). Next, it was incubated for 2 h at -20℃, thawed at room temperature, and centrifuged at 4120 ×*g* and 4℃ for 30 min. The supernatant was removed and filtered through a 0.22-µm filter. Ammonium sulfate was added to the supernatant until the concentration was 40%, then the supernatant was placed at 4℃ for 2 h, centrifuged at 4120 ×*g* for 30 min, and the supernatant was removed. The precipitation was dissolved in PBS (pH 7.4). Then, ammonium sulfate was added to form a final concentration of 35%, and the mixture was placed at 4℃ for 2 h, then centrifuged at 4120 ×*g* for 30 min. The supernatant was discarded and the precipitation was dissolved in PBS (pH 7.4). The concentration of IgY protein was measured photometrically at 280 nm using UV spectra (NanoDrop One, Thermo Fisher Scientific Inc, USA) and was calculated according to the Lambert-Beer law with an extinction coefficient of 1.36 for IgY. The purity of IgY was monitored with SDS-PAGE.

### ELISA testing of IgY binding ability with RBD protein

ELISA was performed to detect the binding of IgY to RBD protein [12]. The SARS-CoV-2 spike RBD-mFc was combined with solid-phase carriers to form the solid-phase conjugate. After washing unbound protein and impurities away, the test product IgYs (3-fold serial dilutions stating from 300 µg/mL) were added to bind with the solid-phase antigen to form the solid-phase immune complex. Next, horseradish peroxidase (HRP)-labeled rabbit anti-chicken IgYs were added. The IgYs on the solid-phase immune complex were combined with an HRP-conjugated antibody. The enzyme catalyzed the substrate that had been added earlier. The OD450 was read by a multi-well spectrophotometer. Data analysis was carried out with GraphPad Prism 8.0.1. The value of EC_50_ was calculated from the best-fit curves for experiment using Prism EC_50_ curve-fitting algorithm.

### Competition ELISA

We used a competition ELISA to evaluate the ability of the IgY to inhibit binding of RBD protein to the human ACE2 [13]. The solid-phase conjugate consisted of SARS-CoV-2 spike RBD-mFc and the solid-phase carrier. The RBD were incubated overnight at 4℃ in high bind 96 well plate. After removing unbound protein and impurities, a serial dilution of purified the IgY and ACE2 (Fc Tag) (Sino Biological Inc., cat. no.: 10,108-H02H) were added to combine with the solid-phase antigen to form the solid-phase immune complex. After incubated for 1 h at 37 ℃, HRP-labeled goat anti-human IgG (FC) was added, and the ACE2-FC on the solid-phase immune complex was combined with HRP-conjugated antibody to catalyze the solid-phase substrate. The inhibition rate was calculated using colorimetry. Data were analyzed using GraphPad Prism 8.0.1.

### Pseudovirus neutralizing assay

The effectiveness of IgY suppression of the SARS-CoV-2 pseudovirus [PSV-Luc-Spike(M)] was measured using luciferase-generated luminescence [14]. 293FT-ACE2 cells were infected with lentivirus expressing SARS-CoV-2 spike protein and luciferase. The light emission was detected by a microporous plate luminometer when the pseudovirus invaded the cells, with the luciferase reacting with the substrate. Lower bioluminescence intensity (relative light unit [RLU]) indicated higher IgY antibody activity against the SARS-CoV-2 pseudovirus. In brief, IgYs were serially diluted, incubated with 100 TCID50/well pseudovirus (1 h at 37℃, in a 5% CO_2_ incubator), and co-cultured with 3 × 10^4^ 293FT-ACE2 cells for 20 h. After incubation, the supernatant was removed and 1×Passive lysis buffer was added at 50 µl/well to lyse the cells. Relative light unit (RLU) of the cell lysate was measured to evaluate luciferase activity. The neutralization percentage was calculated by the formula: Neutralization (%) = (Positive Control RLUs-Sample RLUs) / (Positive Control RLUs-Negative Control RLUs) ×100%. Neutralization titers of the antibodies were presented as 50% maximal inhibitory concentration (EC_50_) which determined by the Reed-Muench method. Data analysis was carried out with Excel 2016.

### Authentic SARS-CoV-2 omicron strain neutralization assay of IgY

We performed an authentic SARS-CoV-2 Omicron BA.2.76 strain virus neutralization assay with anti- SARS-CoV-2 Omicron BA.2.76 strain IgYs in a biosafety level 3 laboratory. The cell culture medium contained a series of anti-SARS-CoV-2 Omicron BA.2.76 strain IgYs from 2-fold serial dilutions starting from 50 µg/mL concentration. After 1-h incubation with the SARS-CoV-2 Omicron BA.2.76 strain virus at 37℃, the IgYs were added to Vero E6 cells, and the cells were cultured for 24 h. Then, the supernatant of the cell culture was discarded and the cells were treated with anhydrous methanol at -20°C for 20 min. The cells were then washed with precooled PBS and incubated with 3% BSA for 2 h at room temperature. Serum from convalescent patients (1:400 dilution) was added as the primary antibody and incubated at 4℃ overnight. The supernatant was discarded and the cells were washed in precooled PBS three times. Then, FITC-labeled anti-human IgG antibody was added for 1-h incubation at room temperature. The supernatant was discarded and the cells were washed with precooled PBS and stained with DAPI (4’, 6-diamidino-2-phenylindole) at room temperature for 10 min. The cells were then washed with precooled PBS once again. The cytopathic phenotype was observed every day and the number of positive cells were counted.

### Large-scale purification of IgYs

The egg yolk of immunized hens was separated by egg yolk sieving, then 8-fold distilled water was added to stir and mix to prepare the mixture at pH 5.0–5.2 (adjusted by 1 M HCl) and held for precipitation at 4℃ overnight. The mixture was centrifuged at 8230 ×*g* for 20 min and the precipitate was discarded. Octanoic acid was slowly added to the collected supernatant to 1% octanoic acid to remove impurities. The mixture was stirred until smooth and the supernatant was collected for ultrafiltration concentration after standing at 4℃ for 2 h and was centrifuged at 4℃ for 30 min at 8230 ×*g*. Diatomite (3%) was added as a filtration aid, and the filtrate was extracted by vacuum filtration with a 0.45-µm mixed cellulose ester membrane (0.09 MPa). A 50,000-molecule interception ultrafiltration membrane was selected, the material was fed at room temperature, and the filtrate was concentrated by ultrafiltration in single-stage batch operation mode to obtain concentrated IgYs, followed by Pasteur disinfection and 0.22-µm membrane filtration.

### Hamster model of SARS-CoV-2 infection

The SARS-CoV-2 strain had been isolated from a patient with laboratory-confirmed COVID-19 by passaging in Vero E6 cells [11]. The virus working stocks were propagated and titrated in Vero E6 cells in the presence of tosyl phenylalanyl chloromethyl ketone (TPCK)-treated trypsin at 2 µg/mL. The stocks were stored at -80 °C prior to experimental infections. All experiments involving infectious viruses were performed in the biosafety level 3 facility of the Second Military Medical University.

The hamsters were randomly classified into three groups (*n* = 6 per group) for the SARS-CoV-2 challenge experiments. Animals were mock-infected in the first group (naïve group). In the second and third groups (control and treatment, respectively), the animals were treated placebo (control group) or the RBD IgY (8.6 mg intraperitoneally and 1.7 mg i.n.) 1 day before challenge, and 1.7 mg i.n. twice daily for five days after challenge. Hamsters in the control and treatment groups were infected i.n. with 8 × 10^4^ TCID50 (median tissue culture infective dose) SARS-CoV-2 diluted in 80 µL Dulbecco’s modified Eagle’s medium as previously described27 [11, 15]. At 4 dpi, the three hamsters in each group were killed and their lungs were obtained for RT-PCR virus load analysis and pathological testing. The weights of the remaining three hamsters in each group were tracked every day for 14 days.

### Hematoxylin and eosin (H&E) staining

The hamster lung tissues were preserved in 4% paraformaldehyde and embedded in paraffin. The tissue Sect. (3 μm) were dewaxed, rehydrated, and underwent routine H&E staining. The tissue sections were examined and imaged under a light microscope. The results were scored semi-quantitatively for alveolar septal thickening and inflammatory cell infiltration, alveolar exudate, and hemorrhage based on the H&E-stained scans [14]. Date were presented as Mean ± SD and were analyzed using unpaired *t* test with GraphPad Prism 8.0.1. *p* < 0.05 was considered statistically significant.

### RNA extraction and RT-qPCR

As previously reported, hamster lung samples were obtained postmortem for viral detection using RT-qPCR [15]. Briefly, total RNA was extracted from the lung tissues using TRIzol (Thermo Fisher Scientific, Shanghai, China). The RNA concentrations and absorbance at 260 nm (A260)/A280 ratio were assessed with a multiplate reader (Synergy 2; BioTek, Shanghai, China). RNA was converted into complementary DNA (cDNA) using a reverse transcription system (Promega, Madison, WI, USA). The cDNA product was used for the following qPCR analysis directly with TB Green Fast qPCR Mix (TaKaRa, Otsu, Japan) and gene-specific primers. Hamster β-actin expression was used for normalization. Additionally, a standard curve was constructed using expression plasmids of the SARS-CoV-2 N gene and hamster β-actin. The SARS-CoV-2 RT-qPCR was quantified as copy numbers. Date were presented as Mean ± SD and were analyzed using unpaired *t* test with GraphPad Prism 8.0.1. *p* < 0.05 was considered statistically significant.

## Results

### RBD-mFc protein antigen production

After the deposit of the newly sequenced SARS-CoV-2 viral genes into the public domain on January 11, 2020, Sino Biological quickly synthesized the spike protein gene and expressed various constructs in HEK293 cells and insect cells. RBD-his, RBD-mFC, S1-his and Spike-ECD were produced in 11 days on January 22, 2020. When the Omicron variant emerged, Sino Biological took only 6-days to produce these viral protein antigens, demonstrating the feasibility of rapid production of viral proteins for immunization.

**Fig. 1:**
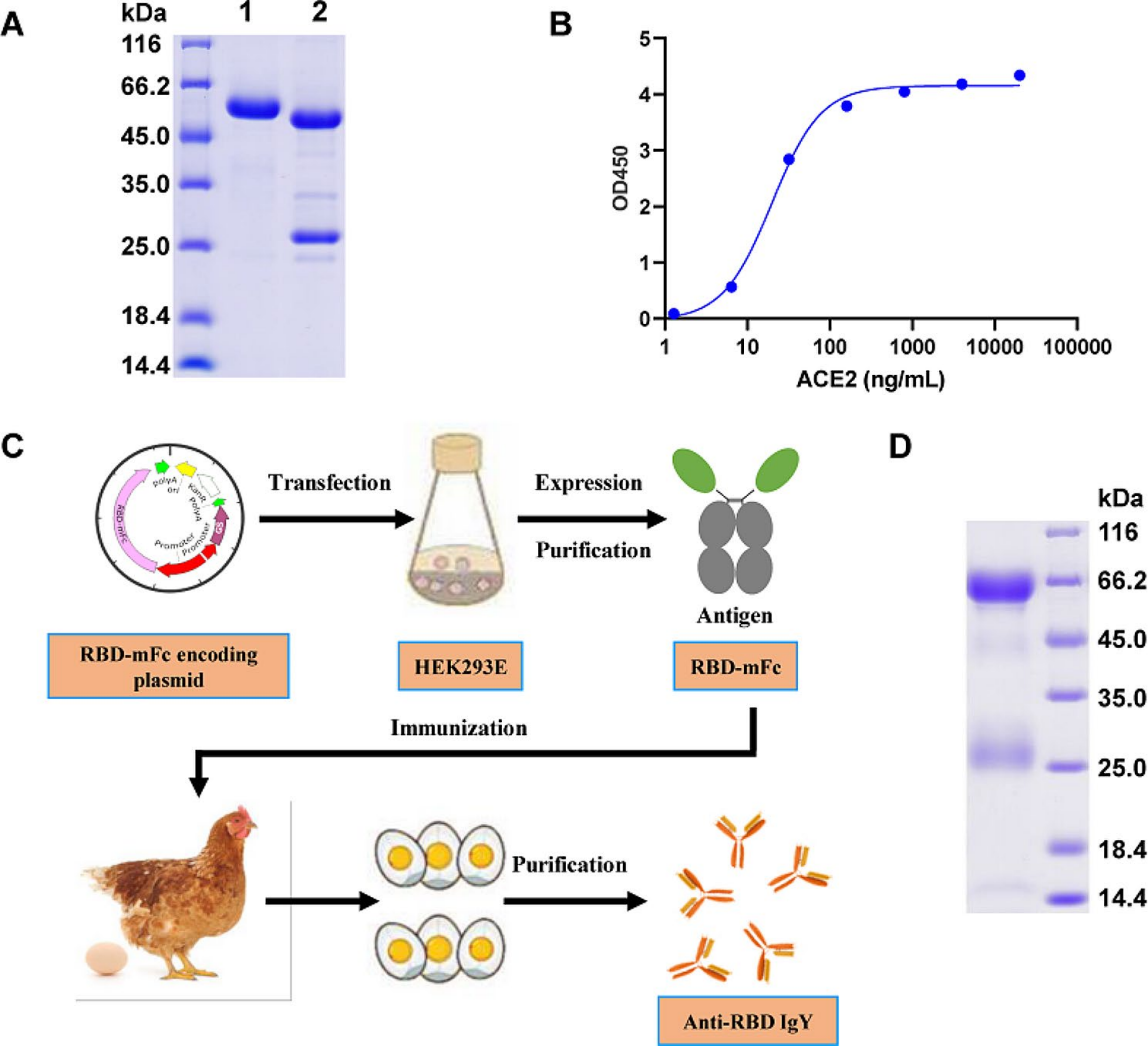
Preparation of anti-SARS-CoV-2 IgY. (**A**) Expression and purification of RBD-mFc proteins. Lane 1: RBD-mFc protein; lane 2: a purified IgG antibody with the heavy chain assigned 50 kDa and the light chain 26 kDa as a molecular control. (**B**) The binding activity of RBD-mFc protein to human ACE2 was determined by ELISA. (**C**) Schematic diagram and workflow for the preparation of RBD IgY. The SARS-CoV-2 RBD-mFC was expressed in HEK293E cells. Hens were immunized by purified RBD-mFc. Anti-RBD IgY were purified from eggs by using 2-step ammonium sulfate precipitation. (**D**) IgY purification. Electrophoresis under reducing conditions demonstrated characteristic IgY banding patterns with molecular weights of 68 kDa and 25 kDa, representing the IgY heavy and light chain, respectively

The viral antigens were used to immunize mice and hen for neutralizing antibody production. RBD-mFc was selected as an example to demonstrate the feasibility of rapid and low cost mass production of polyclonal neutralizing IgY antibodies, due to high expression yield and high purity (> 95%) production with a one-step protein A affinity purification (Fig. 1A). We determined the activity of RBD-mFc protein binding with the ACE2 receptor with enzyme-linked immunosorbent assay (ELISA) (Fig. 1B), which indicated a significantly higher ability to bind ACE2 at a median effective concentration (EC_50_) of 19.8 ng/mL.

### Hen immunization and IgY purification

The procedure of anti-SARS-CoV-2 IgY preparation is described in Fig. 1C. The IgY titer in immunized hens rose continually following the second and fifth booster immunizations on day 10 and 60, respectively (S1 Fig). The eggs collected after the fifth booster immunization on days 57–84 contained high titers of IgY. The titers increased continually post-sixth immunization. The purified IgYs were identified using electrophoresis under reducing conditions (Fig. 1D). Generally, each yolk yielded an average 58.8 mg of IgY.

### IgY characterization in vitro

**Fig. 2:**
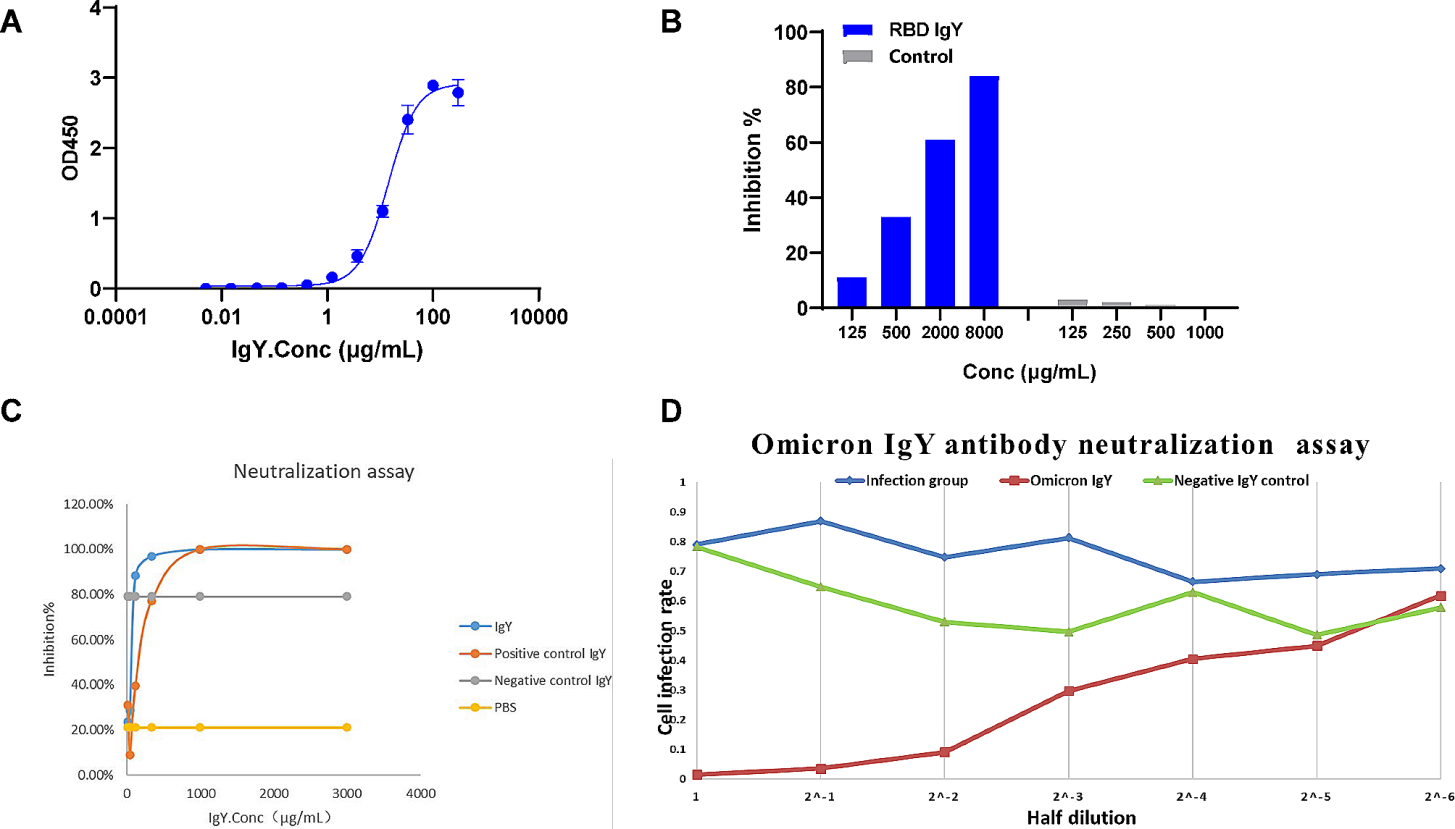
Characterization of IgYin vitro. (**A**) IgY binding activity with RBD. (**B**) IgY inhibition activity of RBD protein binding with ACE2. (**C**) Pseudotype SARS-CoV-2 virus neutralizing assay. (**D**) Authentic SAR-CoV-2 Omicron BA.2.76 strain virus neutralization assay of anti SAR-CoV-2 Omicron Omicron BA.2.76 strain IgY

The purified IgY antibodies were characterized with various analytical methods (S1 Table, Fig. 2). ELISA of the antibody immunoreactivity (Fig. 2A) showed that the relative antigen-binding activity of IgY with RBD was 100% at an EC_50_ of 14.06 µg/mL (R^2^ = 0.9988). The data analysis (Fig. 2B) showed that increasing the IgY concentration prevented the interaction between RBD and ACE2 effectively. We performed pseudoviral infection assays using 293FT-ACE2 cells (Fig. 2C), which showed that IgY against the SARS-CoV-2 pseudovirus at an EC_50_ at 55 µg/mL.

The live authentic viral infection assay was performed in the biosafety level 3 laboratory of the Second Military Medical University, Shanghai, China. The IgYs demonstrated obvious binding with SARS-CoV-2 Omicron BA.2.76 strain (Fig. 2D) and blocked SARS-CoV-2 Omicron BA.2.76 strain infection. Figure 2D shows that 50 µg/mL SARS-CoV-2- Omicron BA.2.76 strain-neutralizing IgY neutralized 99% of SARS-CoV-2 Omicron BA.2.76 strain, which is the first such evidence reported.

### IgY antibodies protected hamster from SARS-CoV-2 challenge

**Fig. 3:**
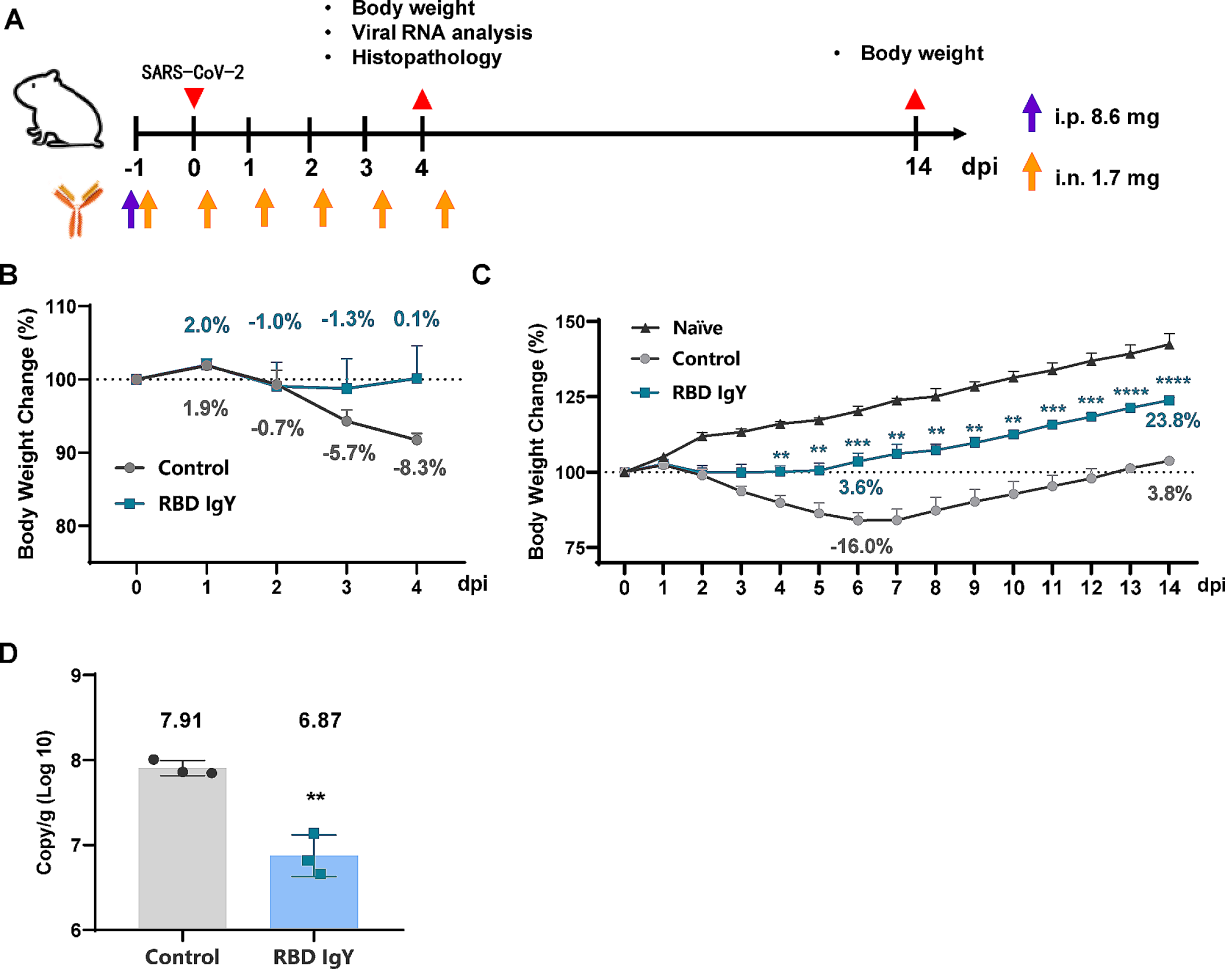
Clinical presentation and viral load in the lungs of Syrian hamsters dosed with RBD IgY following SARS-CoV-2 infection. (**A**) Schematic overview of the animal experiment. (**B**) and (**C**), Hamster body weights were recorded daily at 0–4 dpi (*n* = 3 per group) and 0–14 dpi (*n* = 3 per group). Weight loss was defined as percentage loss from 0 dpi. (**D**) Analysis of the virus load in the lung at 4 dpi (*n* = 3 per group in the control [PBS] group). Comparisons were performed with the control group by unpaired *t* test in GraphPad Prism 8.0.1 each day. Significance levels are **P < 0.01, ***P < 0.001, and ****P < 0.0001

Clinical presentation and viral load in the lungs of Syrian hamsters dosed with RBD IgY following SARS-CoV-2 infection were assayed( Fig. 3A ). Experimental SARS-CoV-2 inoculation via the intranasal (i.n.) routeresulted in transient but significant weight loss in untreated animals as early as 2 days post-inoculation (dpi), which approached 16% weight loss by day 6 dpi and normalized by day 13 dpi (Fig. 3B and C Fig. 3A). Compared to the controls, RBD IgY treatment protected the animals against significant weight loss between day 4 and 14 dpi (Fig. 3B and C). At 4 dpi following IgY antibody administration, reverse transcription–quantitative PCR (RT-qPCR) assessments of lung viral genomic RNA copies demonstrated a 1.04 log 10 (*P* = 0.0023) reduction in the IgY antibody groups, representing a significant reduction in SARS-CoV-2 particles in the lungs (Fig. 3D).

**Fig. 4:**
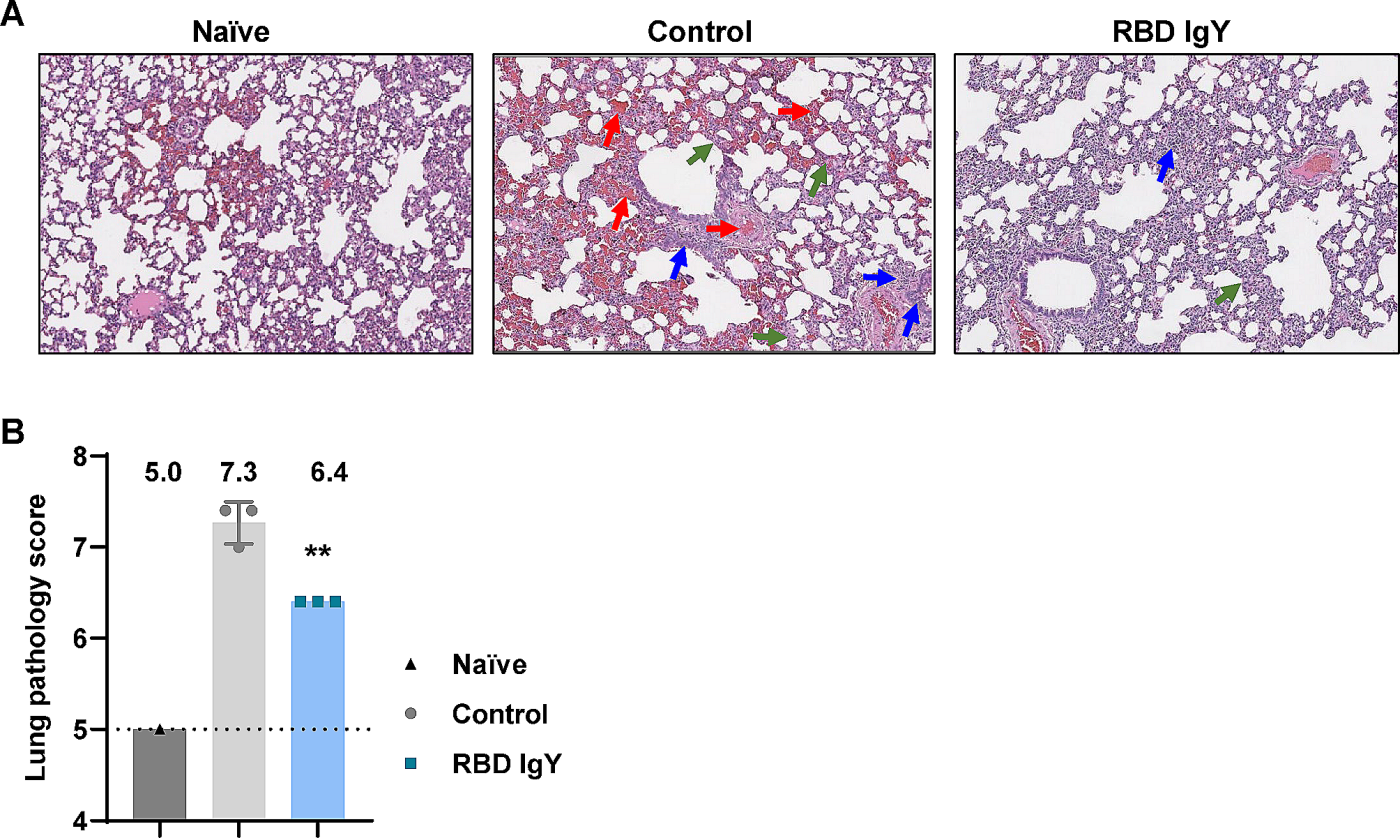
Histopathological analysis of hamsters after SARS-CoV-2 infection. (**A**) Representative histopathology (10×) of the lungs from naïve, control, or RBD IgY-treated hamsters after challenge with SARS-CoV-2 at 4 dpi. The *green arrows* indicate alveolar septal thickening, *blue arrows* indicate inflammatory cell infiltration, *red arrows* indicate bleeding, and exudates in the vascular or alveolar spaces. (**B**) Lung pathology scoring in each group. Comparisons were performed with the control group by unpaired *t* test in GraphPad Prism each day. Significance levels are **P < 0.01

We evaluated lung specimens from the 4 dpi necropsy group and calculated the pathological scores. A naïve hamster was included as a control and presented with mild lesions of alveolar epithelial cells, focal hemorrhage, and inflammatory cell infiltration. These hypercellular areas may represent regions of atelectasis or infection by respiratory commensal [16]. All lungs from the control-treated animals demonstrated similar histological symptoms of pneumonia, including a substantial area of alveolar septal thickening, inflammatory cell infiltration, and bleeding, and exudates in the vascular or alveolar spaces. The RBD IgY-treated animals had significantly reduced lung histological indications (Fig. 4A, B). Infected hamsters that received systemic administration of the SARS-CoV-2 RBD IgY antibodies demonstrated significant reduction of viral load in the lungs and avoided weight reduction and lung pathology.

## Discussion

Vaccination remains the most effective method to control pandemic. With the record-breaking speeds of COVID-19 vaccine development and regulatory authorization or approval, a record number of vaccine doses have been administered. However, a significant percentage of people in developed countries where vaccine supplies are abundant remain unvaccinated, due to various reasons, such as concerns of side effects and lack of long-term safety data. On the other hand, vaccination rate remained very low in developing countries due to availability and affordability issues as well as challenges to meet low-temperature transportation and storage requirements. Most COVID-19 vaccines require 2 intramuscular (i.m.) doses given 3–4 weeks apart. Hence, despite the record speed of vaccine development, it took significant time for mass immunization and for immunized people to develop immunity against the pandemic virus. Furthermore, the rapid emergence of vaccine evading mutation variants outpaces vaccine development, which complicates efforts to achieve herd immunity.

Apparently, despite the great success achieved in COVID-19 vaccine and therapeutic antibody development, there is still a vital need to develop complimentary therapeutics and prophylactics to combat COVID-19 and future emerging pandemic viral agents.

IgYs have functional resemblance to mammalian IgGs [17] and have been studied for more than a century [18]. IgY antibodies demonstrate excellent safety as they do not combine with human Fc receptors or activate mammalian complement components [9], hence do not cause immune responses or antibody-dependent enhancement [19], which could be an advantage for usage in people with immature or impaired immune responses, such as infants and immunocompromised adults [20].

IgY antibodies have outstanding stability during processing or storage at room temperature for 6 months [21], can maintain physiological activity between pH 4 and 11 [22]. IgY antibodies can be produced and purified on a large scale in the context of industrial egg production at low and affordable cost in most countries as no expensive or complicated equipment is needed. These features make it possible for mass production, distribution and application of antiviral IgY antibodies in developing countries.

In this study, we demonstrated that SARS-CoV-2 spike protein antigens can be produced in in 1–2 weeks. The selection of RBD-mFc construct allowed rapid and high yield expression as well as simple one-step affinity purification to achieve high purity antigen for chicken immunization. High titer chicken eggs can be obtained and IgY antibodies can be purified in 2 months, in comparison to much longer time for vaccine and therapeutic monoclonal antibody development.

To date, there has been some research on the potential anti-SARS-CoV-2 effects of IgYs. IgY targeting the full-length S protein, S1 subunit, or RBD have been produced and have demonstrated strong and significant inhibition of RBD–ACE2 binding interactions and variable neutralizing activity against SARS-CoV-2 [23–27]. A Phase 1 study for assessing the safety and tolerability of anti-SARS-CoV-2 S IgY given i.n. to healthy participants has been initiated (NCT04567810). These studies show promise for IgY antibodies as passive immunizers against SARS-CoV-2.

Our study demonstrated feasibility of rapidly generating purified IgY antibodies that neutralized authentic SARS-CoV-2 Omicron BA.2.76 strain. We also assessed the in vivo efficacy of RBD IgY in a Syrian hamster challenge model, which is distinguished by a severe phenotype with weight reduction and specific lung disease. Our findings reveal that high-dose RBD IgY antibodies resulted in not only clinical recovery (as evidenced by the lack of weight reduction), but also a significant reduction in lung viral load and pathology.

Moreover, we produced several IgY formulations, including nasal spray, atomization treatment, eye drops, oral drench, oral paste, or bolus. These types of formulation with other medicines have been approved for individual and pet-related prevention; are easy to use anytime and anywhere; and could become a simple and effective low-cost means for epidemic prevention. Investigation of the SARS-CoV-2 susceptibility of animals that share close relationships with humans has revealed that cats are permissive to infection by airborne transmission [28]. These IgY formulations can also be used on animal pets, which would protect them against SARS-CoV-2 and would aid animal management for controlling COVID-19.

## Conclusion

In this research, we demonstrated proof-of-concept and feasibility of rapid and mass production of neutralizing and in vivo protective polyclonal chicken IgY antibodies within 60 days of obtaining viral antigen gene sequence. No sophisticated facility is required and hence can be set up in a short period of time and the process can be easily implemented in most developing counties to allow low cost and commercial scale production. We believe that this provides an alternative and complementary option for rapid control of newly emerging pandemic prior to and after the successful development of vaccines and therapeutics, and hence warrants further attention and developmental efforts.

### Electronic supplementary material

Below is the link to the electronic supplementary material.

Supplementary Material 1

Supplementary Material 2

Supplementary Material 3

Supplementary Material 4

## Data Availability

All data required to assess the conclusions of the article are present in the article and/or in the supplementary material. The data and materials used in this study are available from the respective authors upon reasonable request.

## References

[1] Wu F, Zhao S, Yu B, Chen YM, Wang W, Song ZG et al. A new coronavirus associated with human respiratory disease in China. Nature. 2020; 579(7798): 265–269. 10.1038/s41586-020-2008-3; PMID: 32015508. 10.1038/s41586-020-2008-3 PMC7094943 32015508

[2] Chen N, Zhou M, Dong X, Qu J, Gong F, Han Y (2020). Epidemiological and clinical characteristics of 99 cases of 2019 novel coronavirus pneumonia in Wuhan, China: a descriptive study. Lancet.

[3] Wu Z, McGoogan JM. Characteristics of and Important Lessons From the Coronavirus Disease 2019 (COVID-19) Outbreak in China: Summary of a Report of 72 314 Cases From the Chinese Center for Disease Control and Prevention. JAMA. 2020; 323(13): 1239–1242. 10.1001/jama.2020.2648; PMID: 32091533. 10.1001/jama.2020.2648 32091533

[4] Zhou F, Yu T, Du R, Fan G, Liu Y, Liu Z et al. Clinical course and risk factors for mortality of adult inpatients with COVID-19 in Wuhan, China: a retrospective cohort study. Lancet. 2020; 395(10229): 1054–1062. 10.1016/S0140-6736(20)30566-3; PMID: 32171076. 10.1016/S0140-6736(20)30566-3 PMC7270627 32171076

[5] Hunter DJ, Abdool Karim SS, Baden LR, Farrar JJ, Hamel MB, Longo DL et al. Addressing Vaccine Inequity - Covid-19 Vaccines as a Global Public Good. N Engl J Med. 2022; 386(12): 1176–1179. 10.1056/NEJMe2202547; PMID: 35196425. 10.1056/NEJMe2202547 35196425

[6] Li D, Sempowski GD, Saunders KO, Acharya P, Haynes BF. SARS-CoV-2 Neutralizing Antibodies for COVID-19 Prevention and Treatment. Annu Rev Med. 2022; 73: 1–16. 10.1146/annurev-med-042420-113838; PMID: 34428080. 10.1146/annurev-med-042420-113838 34428080

[7] Shiraki K, Sato N, Sakai K, Matsumoto S, Kaszynski RH, Takemoto M. Antiviral therapy for COVID-19: Derivation of optimal strategy based on past antiviral and favipiravir experiences. Pharmacol Ther. 2022; 235: 108121. 10.1016/j.pharmthera.2022.108121; PMID: 35121001. 10.1016/j.pharmthera.2022.108121 PMC8806403 35121001

[8] Zarenezhad E, Marzi M. Review on molnupiravir as a promising oral drug for the treatment of COVID-19. Med Chem Res. 2022; 31(2): 232–243. 10.1007/s00044-021-02841-3; PMID: 35002192. 10.1007/s00044-021-02841-3 PMC8721938 35002192

[9] Abbas AT, El-Kafrawy SA, Sohrab SS, Azhar EIA. IgY antibodies for the immunoprophylaxis and therapy of respiratory infections. Hum Vaccin Immunother. 2019; 15(1): 264–275. doi: 10.1080/21645515.2018.1514224; PMID: 30230944. 10.1080/21645515.2018.1514224 PMC6363154 30230944

[10] Lee L, Samardzic K, Wallach M, Frumkin LR, Mochly-Rosen D. Immunoglobulin Y for Potential Diagnostic and Therapeutic Applications in Infectious Diseases. Front Immunol. 2021; 12: 696003. 10.3389/fimmu.2021.696003; PMID: 34177963. 10.3389/fimmu.2021.696003 PMC8220206 34177963

[11] Tai W, He L, Zhang X, Pu J, Voronin D, Jiang S, Du L (2020). Characterization of the receptor-binding domain (RBD) of 2019 novel coronavirus: implication for development of RBD protein as a viral attachment inhibitor and vaccine. Cell Mol Immunol.

[12] Lu, Y., Wang, Y., Zhang, Z., Huang, J., Yao, M., Huang, G., … Wang, W. (2020). Generation of chicken IgY against SARS-COV-2 spike protein and epitope mapping. Journal of immunology research, 2020. 10.1155/2020/9465398 PMC7568776 33134398

[13] Zhu, L., Deng, Y. Q., Zhang, R. R., Cui, Z., Sun, C. Y., Fan, C. F., … Qin, C. F.(2021). Double lock of a potent human therapeutic monoclonal antibody against SARS-CoV-2.National Science Review, 8(3), nwaa297. 10.1093/nsr/nwaa297 PMC7798916 34676096

[14] Sun C, Kong D, Guo E, Zhao J, Jia J, Wang R, Xie L (2023). A Polysaccharide-RBD-Fc-Conjugated COVID-19 vaccine, SCTV01A, showed high immunogenicity and low toxicity in animal models. Vaccines.

[15] Peng H, Ding C, Jiang L, Tang W, Liu Y, Zhao L et al. Discovery of potential anti-SARS-CoV-2 drugs based on large-scale screening in vitro and effect evaluation in vivo. Sci China Life Sci. 2022; 65(6): 1181–1197. 10.1007/s11427-021-2031-7; PMID: 34962614. 10.1007/s11427-021-2031-7 PMC8713546 34962614

[16] Meyer M, Wang Y, Edwards D, Smith GR, Rubenstein AB, Ramanathan P et al. mRNA-1273 efficacy in a severe COVID-19 model: attenuated activation of pulmonary immune cells after challenge. bioRxiv [Preprint]. 2021 [cited 2022 August 18]. https://www.ncbi.nlm.nih.gov/pmc/articles/PMC7852274/

[17] Warr GW, Magor KE, Higgins DA. IgY: clues to the origins of modern antibodies. Immunol Today. 1995; 16(8): 392-8. 10.1016/0167-5699(95)80008-5; PMID: 7546196. 10.1016/0167-5699(95)80008-5 7546196

[18] Klemperer F (1970). Ueber natürliche Immunität und ihre Verwerthung für die Immunisirungstherapie. Archiv Für Experimentelle Pathologie Und Pharmakologie.

[19] Kovacs-Nolan J, Mine Y. Egg yolk antibodies for passive immunity. Annu Rev Food Sci Technol. 2012; 3: 163 – 82. 10.1146/annurev-food-022811-101137; PMID: 22136128. 10.1146/annurev-food-022811-101137 22136128

[20] Zhang X, Calvert RA, Sutton BJ, Doré KA. IgY: a key isotype in antibody evolution. Biol Rev Camb Philos Soc. 2017; 92(4): 2144–2156. 10.1111/brv.12325; PMID: 28299878. 10.1111/brv.12325 28299878

[21] Nilsson E, Stålberg J, Larsson A. IgY stability in eggs stored at room temperature or at + 4°C. Br Poult Sci. 2012; 53(1): 42 – 6. doi: 10.1080/00071668.2011.646951; PMID: 22404803. 10.1080/00071668.2011.646951 22404803

[22] Lee KA, Chang SK, Lee YJ, Lee JH, Koo NS. Acid stability of anti-Helicobacter pyroli IgY in aqueous polyol solution. J Biochem Mol Biol. 2002; 35(5): 488 – 93. 10.5483/bmbrep.2002.35.5.488; PMID: 12359091. 10.5483/bmbrep.2002.35.5.488 12359091

[23] Bao L, Zhang C, Lyu J, Yi P, Shen X, Tang B et al. Egg yolk immunoglobulin (IgY) targeting SARS-CoV-2 S1 as potential virus entry blocker. J Appl Microbiol. 2022; 132(3): 2421–2430. 10.1111/jam.15340; PMID: 34706134. 10.1111/jam.15340 PMC8657347 34706134

[24] Frumkin LR, Lucas M, Scribner CL, Ortega-Heinly N, Rogers J, Yin G et al. Egg-Derived Anti-SARS-CoV-2 Immunoglobulin Y (IgY) With Broad Variant Activity as Intranasal Prophylaxis Against COVID-19. Front Immunol. 2022; 13: 899617. 10.3389/fimmu.2022.899617; PMID: 35720389. 10.3389/fimmu.2022.899617 PMC9199392 35720389

[25] Kadiyala G, Iyer S, Meher K (2021). Preparation of ingestible antibodies to neutralize the binding of SarsCoV2 RBD (receptor binding domain) to human ACE2 receptor. bioRxiv.

[26] Lu Y, Wang Y, Zhang Z, Huang J, Yao M, Huang G et al. Generation of Chicken IgY against SARS-COV-2 Spike Protein and Epitope Mapping. J Immunol Res. 2020; 2020: 9465398. 10.1155/2020/9465398; PMID: 33134398. 10.1155/2020/9465398 PMC7568776 33134398

[27] Wei S, Duan S, Liu X, Wang H, Ding S, Chen Y et al. Chicken Egg Yolk Antibodies (IgYs) block the binding of multiple SARS-CoV-2 spike protein variants to human ACE2. Int Immunopharmacol. 2021; 90: 107172. 10.1016/j.intimp.2020.107172; PMID: 33191178. 10.1016/j.intimp.2020.107172 PMC7608017 33191178

[28] Shi J, Wen Z, Zhong G, Yang H, Wang C, Huang B et al. Susceptibility of ferrets, cats, dogs, and other domesticated animals to SARS-coronavirus 2. Science. 2020; 368(6494): 1016–1020. 10.1126/science.abb7015; PMID: 32269068. 10.1126/science.abb7015 PMC7164390 32269068

