# Supplementary material for: Rapid development and mass production of SARS-CoV-2 neutralizing chicken egg yolk antibodies with protective efficacy in hamsters
Source: Biol Res. 2024 May 6;57:24. doi: 10.1186/s40659-024-00508-y (PMC11071260; doi:10.1186/s40659-024-00508-y)
Supplement: Supplementary file 1 — Supplementary Material 1 [file 40659_2024_508_MOESM1_ESM.doc]

**Supplemental Figures**


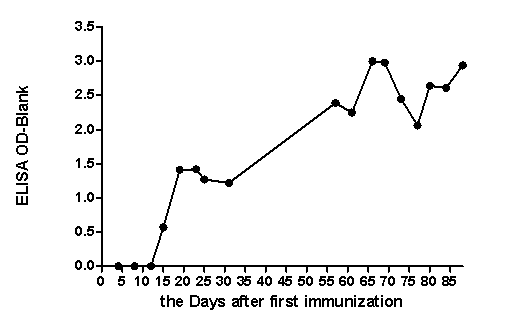


**Fig. S1. The IgYs titer detection of egg yolk supernatant.** Eggs were collected from immunized hens after first immunization, and analyzed by ELISA against RBD-mFC immunogen.

**Table. S1. Quality control data of IgYs**

| Immunogen | Proteinconcentration（mg/mL） | SDS-PAGE  purity（%） | SEC-HPLC(%) | EC50  (μg/mL) | InhibitionACE2-Fc | Titer of IgYs to  RBD | Pseudovial neutralizing assay  EC50 （ng/mL） |
| --- | --- | --- | --- | --- | --- | --- | --- |
|  |  |  |  | With  RBD | Compete with RBD |  |  |
| RBD-mFc | 19.06 | 77.7 | 91.9% | 0.19 | competion | 1:576000 | 8071 |
| Negative control | 1.99 | / | / | Non | Non | / | / |
