# Supplementary figures and images for: Rapid development and mass production of SARS-CoV-2 neutralizing chicken egg yolk antibodies with protective efficacy in hamsters
Source: Biol Res. 2024 May 6;57:24. doi: 10.1186/s40659-024-00508-y (PMC11071260; doi:10.1186/s40659-024-00508-y)

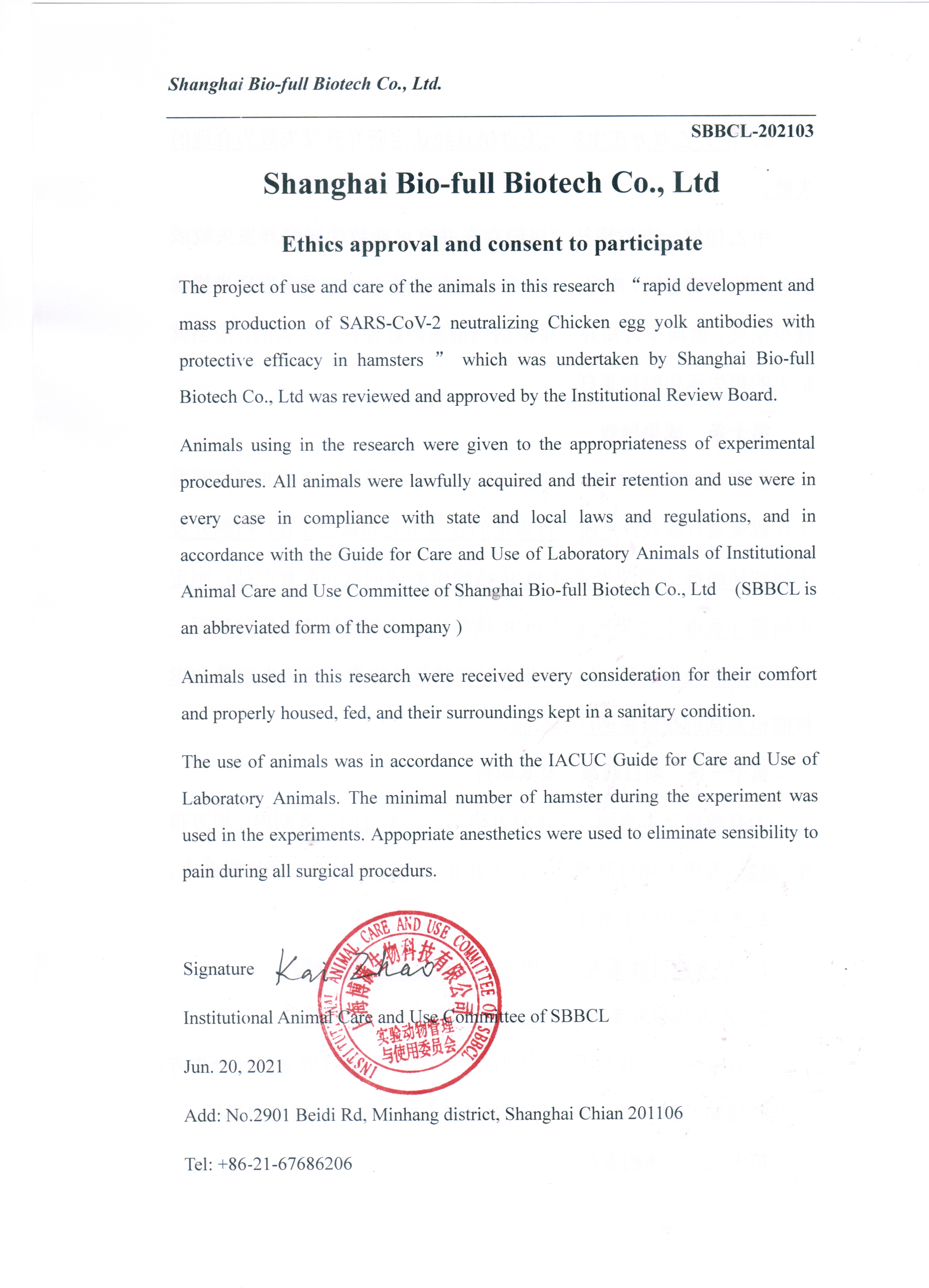

Supplement: Supplementary file 2 — Supplementary Material 2 [file 40659_2024_508_MOESM2_ESM.jpg]
