# Supplementary material for: Rapid development and mass production of SARS-CoV-2 neutralizing chicken egg yolk antibodies with protective efficacy in hamsters
Source: Biol Res. 2024 May 6;57:24. doi: 10.1186/s40659-024-00508-y (PMC11071260; doi:10.1186/s40659-024-00508-y)
Supplement: Supplementary file 4 — Supplementary Material 4 [file 40659_2024_508_MOESM4_ESM.docx]

Subject: Your submission to **Biological Research** - BRES-D-23-00701 - [EMID:f1b9ed5401139911]

BRES-D-23-00701

Rapid development and mass production of SARS-CoV-2 neutralizing Chicken egg yolk antibodies with protective efficacy in hamsters

Binan Zhao; Haoran Peng; Yanjing Zhang; Jie Zhang; Desheng Kong; Sai Cao; Yan Li; Dan Yang; Chuanwen Sun; Xinyi Pu; Ping Zhao; Yan Xu; Kai Zhao; Liangzhi Xie

Biological Research

Dear Prof. Zhao,

Your manuscript 'Rapid development and mass production of SARS-CoV-2 neutralizing Chicken egg yolk antibodies with protective efficacy in hamsters' (BRES-D-23-00701) has been assessed by our reviewers. Although it is of interest, we are unable to consider it for publication in its current form. The reviewers have raised a number of points which we believe would improve the manuscript and may allow a revised version to be published in Biological Research.

Their reports, together with any other comments, are below. Please also take a moment to check our website at https://www.editorialmanager.com/bres/ for any additional comments that were saved as attachments.

If you are able to fully address these points, we would encourage you to submit a revised manuscript to Biological Research. Once you have made the necessary corrections, please submit online at: https://www.editorialmanager.com/bres/

If you have forgotten your username or password please use the 'Send Login Details' link to get your login information. For security reasons, your password will be reset.

The due date for submitting the revised version of your article is 20 Mar 2024.

Please include a cover letter with a point-by-point response to the reviewer comments, describing additional experiments that were carried out and including a detailed rebuttal of any criticisms or requested revisions that you disagreed with. Please also ensure that your revised manuscript conforms to the journal style, which can be found in the Instructions for Authors on the journal homepage.

Please note, if your manuscript is accepted you will not be able to make any changes to the authors, or order of authors, of your manuscript once the editor has accepted your manuscript for publication. If you wish to make any changes to authorship before you resubmit your revisions, please reply to this email and ask for a 'Request for change in authorship' form which should be completed by all authors (including those to be removed) and returned to this email address. Please ensure that any changes in authorship fulfil the criteria for authorship as outlined in BioMed Central's editorial policies (http://www.biomedcentral.com/about/editorialpolicies#authorship).

Once you have completed and returned the form, your request will be considered and you will be advised whether the requested changes will be allowed.

By resubmitting your manuscript you confirm that all author details on the revised version are correct, that all authors have agreed to authorship and order of authorship for this manuscript and that all authors have the appropriate permissions and rights to the reported data.

Please be aware that we may investigate, or ask your institute to investigate, any unauthorised attempts to change authorship or discrepancies in authorship between the submitted and revised versions of your manuscript.

I look forward to receiving your revised manuscript soon.

Best wishes,

Biological Research

https://biolres.biomedcentral.com/

**Reviewer reports:**

**Reviewer #1:** The article proposes a therapy based on neutralizing antibodies based on IgY against SARS-CoV-2.

The article demonstrates that IgY purified from chicken eggs can be used as a preventive, but non-paleative, therapy against SARS-CoV-2. Since its effectiveness is analyzed before infection and not after infection.

The comments are:

- Page 3, Line 6, and Page 12, line 30: "Error! Reference source not found".

- Page 3, line 42-53. Need references.

- Page 3, line 4-23, need references.

-Figure 1A. Please, add whole expression from Hek293. Also, molecular weight seems irregular, please add more controls. Same aa figure 1D. add more controls used to determine the purification process of IgY.

**Response:** Thanks for your suggestions. The expression supernatant from HEK293 has been used up, we added a purified IgG antibody with the heavy chain assigned 50 kDa and the light chain 26 kDa to SDS-PAGE as a molecular control in Fig 1A. Fig 1D used the same protein marker like Fig 1A, thus no new controls added.

-Figure 2A. How many replicates were made? please add standard deviation.

**Response:** Thanks for the detailed suggestion. We did three replicates and standard deviation has been added in Figure 2A.

- Page 4, line 35. "We performed pseudoviral infection assays using 293FT-ACE2 cells (Fig 2C), which showed that IgY against the SARS-CoV-2 pseudovirus at an EC50 at 55 μg/mL." In Figure 2B, it is identified that between 500 and 2000 ug/mL, a value of 50% inhibition is reached. How can this be possible?

**Response:** We think it was caused by different methods. Fig 2C showed neutralizing activities of IgY against SARS-CoV-2 with an EC50 value of 55 μg/mL by cell-based pseudoviruses neutralization assay. Whereas Fig 2B demonstrated IgY compete with recombinant ACE2 for binding to RBD with an EC50 value between 500 and 2000 ug/mL by the enzyme-linked immunosorbent assay. Similar to other publications (National Science Review, Volume 8, Issue 3, March 2021, nwaa297, https://doi.org/10.1093/nsr/nwaa297), there was approximately a 10-fold difference in EC50 between these two methods.

- Please add information on how the EC50 calculation was carried out.

**Response:** Thanks for your careful review. We have added EC_50_ calculate information in methods section in detail.

- It is necessary to add information related to the statistical analysis, and what softwares were used.

**Response:** Thanks for your suggestion. We performed statistical analysis in Fig 3 & Fig 4 by unpaired *t* test in GraphPad Prism 8.0.1, which described in the figure legend. We have added this information in methods section for better understanding.

- In the materials and methods section, it is stated that 6 animals per group were used to challenge animals with SARS-CoV-2. However, in Figure 4 only 3 animals per group were analyzed.

**Response:** In challenge experiments, 6 animals per group were used to challenge SARS-CoV-2. At 4 dpi, the three hamsters in each group were killed and their lungs were obtained for RT-PCR virus load analysis and pathological testing. The weights of the remaining three hamsters in each group were tracked every day for 14 days. Thus hamster body weights were recorded daily at 0–4 dpi (n = 3 per group, killed at 4 dpi for virus load analysis and pathological testing, Fig 3B & 3D) and 0–14 dpi (n = 3 per group, Fig 3C).

- There are several analyzes that show a single analysis. In this context, of the 10 vaccinated hens, what was the criterion for using IgY from one of ten hens. Or was a pool of the 10 hens made?

**Response:** IgY was purified from a pool of the 10 hens.

**Reviewer #2**: -This study describes the protective activity of the purified IgY antibodies targeting RBD-mFc protein against COVID-19 in experimentally infected n hamsters. The work is well structured, well planned and the research is competently carried out, the methodology was quite adequate to the research, but the novelty of the research is very poor because previous studies was covered this topic. Specific comments on the manuscript are listed below:

- Please provide citation for each technique in the methodology section.

**Response:** Thanks for your careful review. We have added citations for each technique in the methodology section.

- The method Described ELISA testing of IgY binding ability with RBD protein, Competition ELISA, Pseudovirus neutralizing assay was very brief, please provide further details for each.

**Response:** Thanks for the detailed suggestion. We have added detailed methods and references.

- The authors may justify how the dose optimized for the present investigation in the materials and methods (either based on available literature or previous study). If from the others study should be supported with reference.

**Response:** We made some adjustments based on the concentration used in the other study. The supported reference has been added.

-Method of determination of the IgY concentration and the purity need to be elucidated in the manuscript.

**Response:** Thanks for your suggestion. IgY antibody concentration and purity detection methods have been added in the “IgY production and purification” section.

-Resolution of the figures need to be improved.

**Response:** Thanks for your suggestion. We have improved resolution of the figures.

-In results, figures should be highlighted with arrow mark about what kind of changes have been noted in the staining slides (histopathological slides) for better understanding.

**Response:** Thanks for the detailed suggestion. Changes like alveolar septal thickening, inflammatory cell infiltration, bleeding, and exudates in the vascular have been highlighted with different colored arrows.

If improvements to the English language within your manuscript have been requested, you should have your manuscript reviewed by someone who is fluent in English. If you would like professional help in revising this manuscript, you can use any reputable English language editing service. We can recommend our affiliates Nature Research Editing Service (http://bit.ly/NRES-LS) and American Journal Experts (http://bit.ly/AJE-LS) for help with English usage. Please note that use of an editing service is neither a requirement nor a guarantee of publication. Free assistance is available from our English language tutorial (https://www.springer.com/gb/authors-editors/authorandreviewertutorials/writinginenglish) and our Writing resources (http://www.biomedcentral.com/getpublished/writing-resources). These cover common mistakes that occur when writing in English.

This letter contains confidential information, is for your own use, and should not be forwarded to third parties.

Recipients of this email are registered users within the Editorial Manager database for this journal. We will keep your information on file to use in the process of submitting, evaluatin
